# A small molecule targeting glutathione activates Nrf2 and inhibits cancer cell growth through promoting Keap-1 *S*-glutathionylation and inducing apoptosis†

**DOI:** 10.1039/c7ra11935f

**Published:** 2018-01-03

**Authors:** LiHong Wang, GuoJing Qu, YuanDi Gao, Le Su, Qing Ye, Fan Jiang, BaoXiang Zhao, JunYing Miao

**Affiliations:** Shandong Provincial Key Laboratory of Animal Cells and Developmental Biology, School of Life Science, Shandong University Jinan 250100 China miaojy@sdu.edu.cn +86 531 88565610 +86 531 88364929; The Key Laboratory of Cardiovascular Remodeling and Function Research, Chinese Ministry of Education, Chinese Ministry of Health, Qilu Hospital, Shandong University Jinan 250012 China; Institute of Organic Chemistry, School of Chemistry and Chemical Engineering, Shandong University Jinan 250100 China

## Abstract

The level of glutathione (GSH) is increased in many cancer cells. Consuming intracellular GSH by chemical small molecules that specifically target GSH is a new strategy to treat cancer. Recently, we synthesized and proved that a new compound 2-(7-(diethylamino)-2-oxo-2*H*-chromen-3-yl)cyclohexa-2,5-diene-1,4-dione (PBQC) could target to and consume intracellular GSH specifically, but, it is not clear if PBQC can affect cancer cell growth and the activity of the nuclear factor-erythroid 2-related factor 2 (Nrf2) which is a key factor involved in regulation of cancer cell growth. In this study, we addressed these questions. We found that PBQC suppressed cancer cell growth through increasing the activity of Nrf2, while it did not inhibit normal vascular endothelial cell growth. Furthermore, we demonstrated that PBQC can cause Keap-1 protein *S*-glutathionylation and promote Nrf2 nuclear translocation as well as the expression of pro-apoptosis genes. As a result, the cancer cells underwent apoptosis. Here, we provide a new Nrf2 activator, PBQC that can promote the expressions of pro-apoptosis genes downstream Nrf2. The data suggest that PBQC is a potential lead-compound for development of new anti-cancer drugs.

## Abstract 2

The level of glutathione (GSH) is increased in many cancer cells.

## Graphical abstract

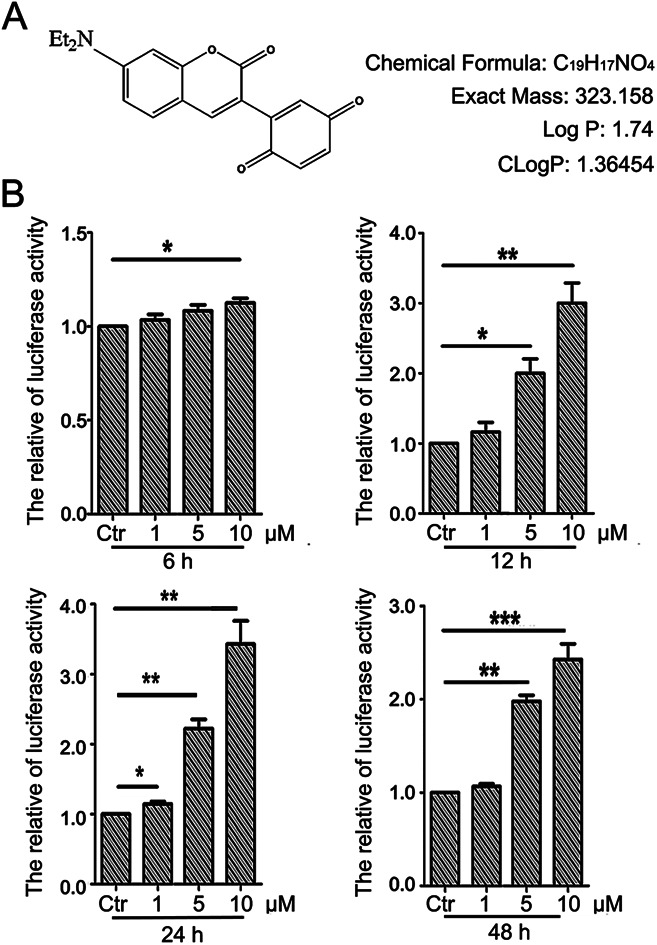

## 1. Introduction

The level of GSH is relatively high in many cancer cells such as lung cancer, breast cancer, pancreatic cancer and leukemia.^1–4^ In addition, it has been demonstrated that the anti-apoptosis feature of cancer cells is related to the increase of the intracellular GSH level.^5^ Several reports have shown that decreasing intracellular GSH content activates various apoptosis related enzymes.^6,7^ Therefore, consuming GSH is becoming a new strategy for anti-tumor therapy.^8,9^

The nuclear factor erythroid-2 related factor 2 (Nrf2) is one of the nuclear transcription factors of CNC (cap-‘n’-collar) family, which includes a highly conserved sequence, basic region-leucine zipper (b-ZIP) proteins.^10^ Nrf2 can interact with the anti-oxidant response element (ARE) and positively regulate the gene expression of detoxifying enzyme and anti-oxidant enzymes that protect cells against oxidative and electrophilic stress.^11,12^ In recent years, Nrf2 has received more and more attention as one of the drug targets for cancer prevention in academic field.^13–15^ Under normal conditions, Keap-1 and Nrf2 are bound in the cytoplasm, in which the activity of Nrf2 is limited. But, the accumulation of Nrf2 in the nucleus of tumor would not only cause glutathione levels increasing but also lead to relevant detoxification enzyme and drug outflow pump gene expression increasing. Then tumor cell metabolism will be damaged and the proliferation will be abnormal.^16,17^ Thus, inhibiting Nrf2 is a normal strategy for the effective treatment of cancer.^18^ Therefore, whether the activated state of the Nrf2 signaling pathway is normal or not determines its double roles in cancer.^19^ Previously, some studies have indicated that Nrf2 inhibitors can interrupt the signaling pathway of Nrf2–ARE. Leading to the increase of chemotherapy sensitivity and inhibition of tumor cells growth.^20–24^ But, recently, studies have shown that Nrf2 activators are also capable of inducing cancer cell apoptosis.^25,26^ This new discovery will provide another new vision for the treatment of the tumor. Now, although there are a large amount of Nrf2 activators, few can promote cell apoptosis. Thus, screening for drugs that activate Nrf2 and promote cell apoptosis has become a new idea for treating tumors.

*S*-Glutathionylation is a specific post-translational modification of protein cysteine residue, in which glutathione is reversibly bound to a thiol group (PSH), resulting in *S*-glutathionylated protein (PSSG).^27,28^ Under normal physiological conditions, *S*-glutathionylation goes on all the time, but this process has been demonstrated to be promoted under the condition of oxidative/nitrosated stress.^27,28^ Keap1 is an extremely thiol-rich protein with a large number of cysteine residues, some of which are predicted to be highly reactive.^29,30^ Recently, it has been reported that the cysteine residues in Keap1 are modified by *S*-glutathionylation and the expression of Nrf2 is significantly increased after GSH depletion.^27^ At the same time, the pro-oxidant increases Keap-1 *S*-glutathionylation and Nrf2 activation subsequently.^31^

In the previous study, we synthesized and proved that a new compound 2-(7-(diethylamino)-2-oxo-2*H*-chromen-3-yl)cyclohexa-2,5-diene-1,4-dione (PBQC) could target to and consume intracellular GSH specifically. We hypothesize that PBQC may affect the activity of Nrf2 and inhibit cancer cell growth. And we demonstrated this hypothesis in this study. We found PBQC could up-regulate the Nrf2 activity as well as inhibit the cancer cell growth. PBQC caused Keap-1 protein *S*-glutathionylation, promoted Nrf2 nuclear translocation and the expression of pro-apoptosis genes. As a result, the cancer cells underwent apoptosis. This discovery may provide a new strategy which is different from previous methods for tumor treatment.

## 2. Materials and methods

### 2.1. Synthesis of PBQC

**Fig. 1:**
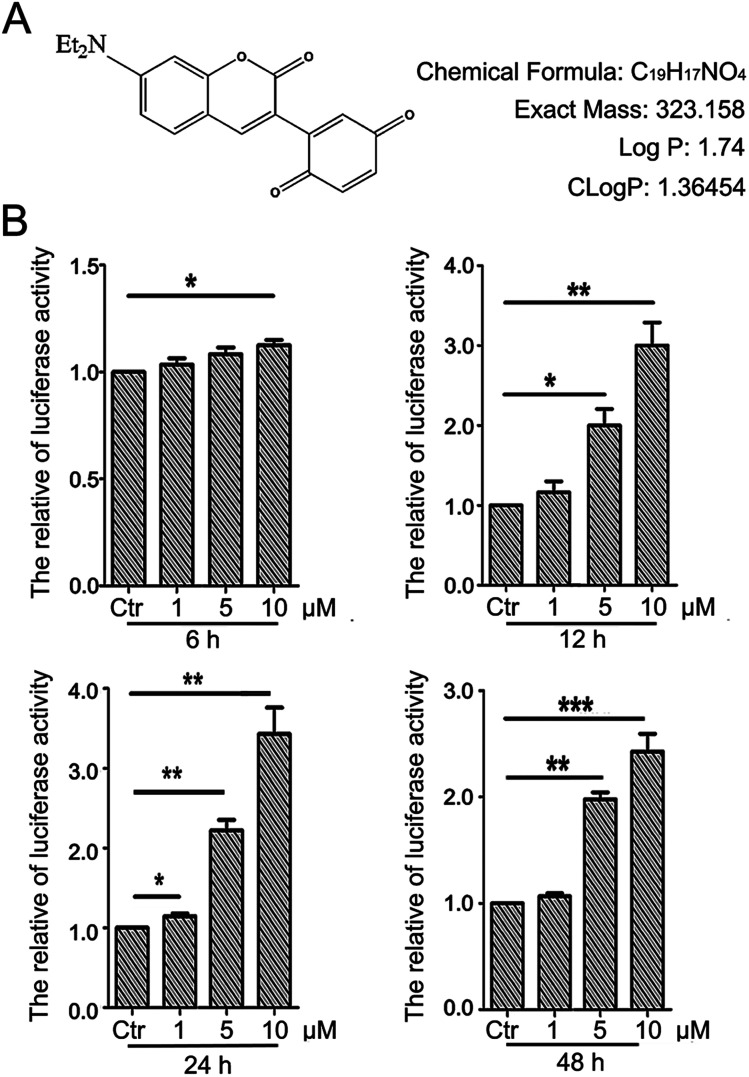
PBQC up regulated Nrf2 activity. (A) PBQC molecular structure is shown. (B) HeLa cells were stably transfected with ARE–luciferase reporter gene, and incubated Luci–HeLa cells with 0.1% DMSO (control) or PBQC at 1, 5, 10 μM for 6, 12, 24 and 48 h. The cell viability was analyzed by SRB assay and Luci–HeLa cells were lysed and luciferase activities were measured, setting the control group activity to 1. (*p < 0.05, **p < 0.01, ***p < 0.001, *vs.* control, *n* = 3).

The detailed procedure for the synthesis and structures of 2-(7-(diethylamino)-2-oxo-2*H*-chromen-3-yl)cyclohexa-2,5-diene-1,4-dione (PBQC) (Fig. 1A) were described in our previous reported.^32^ We designed and synthesized PBQC by PET process based on coumarin and quinone.

### 2.2. Cell culture

HeLa cells and luciferase HeLa cells were cultured in Dulbecco's Modified Eagle Medium-High glucose (DMEM-HG) (Gibco) supplemented with 10% calf bovine serum (HyClone), A549 cells were cultured in RPMI Medium 1640 (Gibco) supplemented with 10% calf bovine serum (HyClone). U87 cells were cultured in Dulbecco's Modified Eagle Medium-High glucose (DMEM-HG) (Gibco) supplemented with 10% fetal bovine serum (HyClone). HeLa cells stably transfected with a luciferase-based Nrf2 reporter plasmid were cultured in Dulbecco Modified Eagle Medium (DMEM, Gibco, 12800-058) with 10% new born calf serum. Human umbilical vein endothelial cells (HUVEC) were cultured in Medium 199 (M199) (Gibco) supplemented with 10% fetal bovine serum (HyClone) at 37 °C in a humidified incubator with 5% CO_2_. In the process of the whole experiment, the cell's density was around 90% before doing all experiment, dissolved plump cells with 0.25% or 0.05% trypsin (Sangon Biotech) in phosphate buffered saline (PBS), the decentralized cells were seeded onto 6-well plates, 12-well plates, 24-well plates, 96-well plates or other appropriate dishes at a density of 20 000 cells per ml pre-incubated 12 h before adding the compound PBQC. HeLa cells and A549 cells were purchased from the ATCC (American Type Culture Collection), U87 cells were provided by Bing Yan (Institute of Analytic Chemistry, School of Chemistry and Chemical Engineering, Shandong University, Jinan, China), and luciferase HeLa cells were provided by Qing Ye and Fan Jiang (The Key Laboratory of Cardiovascular Remodeling and Function Research, Chinese Ministry of Education and Chinese Ministry of Health, Qilu Hospital, Shandong University, Jinan, China). Human umbilical vein endothelial cells (HUVEC) were provided by People's Liberation Army 456 Hospital, Jinan.

### 2.3. Luciferase assay

Luciferase HeLa cells that can stably express an Nrf2-responsive reporter plasmid (pGL4-3 × ARE) were seeded into 96-well plates at the density of 40 000 cells per ml and consistently cultured for 12 h. About twelve hours, we respectively incubated luciferase HeLa cells with PBQC (concentration, 0, 1, 5 and 10 μM) for 6, 12, 24 and 48 h. And then we added and collected the reporter gene cell lysis solution (RG-005-1), examined the luciferase activity by Lucifer Reporter Gene Assay Kit (RG005, Beyotime, Shanghai). Finally, the luciferase activity was normalized to cell viability which assessed by SRB assay.^33^

### 2.4. Cell morphological observation

HeLa cells, A549 cells, U87 cells and HUVECs were seeded into 24-well plates and treated with 0.1% DMSO as a vehicle control or 1, 5 and 10 μM PBQC for 6, 12, 24 and 48 h, taken the microscopic photographs (200×) under the inverted phase contrast microscope (Nikon, Japan).

### 2.5. Cell viability assay (SRB)

According to the relevant reports, HeLa cells, A549 cells, U87 cells and HUVECs were seeded into 96-well plates. After 12 h, cells were respectively treated with 0.1% DMSO as a relevant control or compound PBQC (concentration, 1, 5 and 10 μM) for specified time durations. And then cell viability was evaluated by sulforhodamine B (SRB) assay, according to the method of Skehan. In short, discarded the original medium and added 100 μL of cold 10% trichloroacetic acid (TCA) into the 96-well plates to fix cells and incubated for 1 h at 4 °C. Poured off the supernatant and washed the cell five times with ultrapure water. After drying the 96-well plates at room temperature, added 50 μL of 0.4% (w/v) SRB solution in 1% acetic acid into each hole and shook 10 min on microtiter plate shaker. And then washed the 96-well plates five times with 1% acetic acid and subsequently added 100 μL of 10 mM unbuffered Tris base (pH = 10.5) to dissolve the original dye after again dried the 96-well plates at room temperature. Shook 10 min on microtiter plate shaker, and finally measured the light absorption at the wavelength of 540 nm used a SpectraMAX190 microplate spectrophotometer (GMI Co, USA).

### 2.6. Co-immunoprecipitation

HeLa cells were lysed in IP buffer (Beyotime, P0013). And the main components of IP buffer (without inhibitor) are 20 mM Tris (pH 7.5), 150 mM NaCl, 1% Triton X-100, without protease, phosphatase and other inhibitors. The lysates were pre-cleared with protein A/G agarose beads (Beyotime, P2012) for 1 h at 4 °C. After centrifugation (3000 rpm), the supernatant was collected and incubated with 10 μL specific antibodies or normal corresponding IgG, then with protein A/G beads overnight at 4 °C. The beads were rinsed with IP buffer 3 times and eluted with 2× SDS loading buffer. The immunoprecipitated proteins were detected by western blot assay.^34^

### 2.7. Immunofluorescence microscopy

For the next immunofluorescence assay, we seeded HeLa cells (20 000 cells per ml) into confocal dishes (20 mm) (SPL, Korea) pre-incubated 12 h and used PBQC to treat cell. After treatment, discarded the culture medium and rinsed the cells gently with 1× PBS, then the cells were fixed in 4% paraformaldehyde for 15 min, after washing three times with 1× PBS, permeated cells with 0.1–0.2% Triton X-100 for 5 min, then washed and blocked with donkey serum (1 : 30 dilution in 0.1 M PBS) for 20 min at room temperature. Discarded the enclosed liquid, incubated cells with primary antibody (1 : 100 dilution) overnight at 4 °C, then with Alexa 488 nm or 546 nm labeled species-specific secondary antibodies (1 : 200 dilution in 0.1 M PBS) for 60 min at 37 °C. Finally, rinsed and stained cells with PI, and monitored by a confocal laser scanning microscope (Zeiss, Germany). The ratio of Nrf2 nuclear translocation was calculated by ImageJ. We only collected the green fluorescence in the nucleus by ImageJ and quantified by GraphPad Prism 5.

### 2.8. Detecting ROS levels

HeLa cells were seeded into 6-well plates at the density of 20 000 cells per ml and consistently cultured for 12 h, then incubated with PBQC (0, 1, 5 and 10 μM) for 6 h or 12 h. Then added 5 μM fluorescent probe DCHF and incubated for 30 min.^26^ Washed 3 times with PBS and underwent imaging measurement by an Olympus BH-2 fluorescence microscope (Nikon, Japan). Finally, the fluorescence intensity was quantified by ImageJ software.

### 2.9. RT-PCR analysis

HeLa cells (20 000 cells per ml) were pretreated with or without the compound PBQC for the given time. Then, RT-PCR analysis of Nrf2, HO-1, GCLC, Bcl-2, Bax, p53 and β-actin were implemented. The total RNA of HeLa cells were extracted and isolated using TRIzol reagent (Life Technologies), and an amount of 1000 ng of total RNA was reverse transcriped using a PrimeScript RT reagent kit with a gDNA Eraser (DRR047A, Takara). Synthesized complementary DNA was augmented by PCR utilizing a 2× EasyTaq PCR SuperMix (Transgen Biotech).

### 2.10. LDH assay

Cells were seeded into 6-well plates at a density of 20 000 cells per ml. Cell culture medium was collected after 48 h treatment with compound PBQC (10 μM) or 0.1% DMSO as a relevant control. LDH assay was detected by using a Lactate Dehydrogenase (LDH) kit (Nanjing Jiancheng Co, China), according to the manufacturer's instructions.^35^ Briefly, prepared treated samples as well as standard samples at different concentrations. Added reaction mix and measured fluorescence (Ex/Em = 535/587 nm) in a kinetic mode at 37 °C 10–30 min.

### 2.11. Hoechst 33258 staining

HeLa cells were seeded into 24-well plates at a density of 20 000 cells per ml. After treating cells with 0.1% DMSO (as control) or compound PBQC (1, 5 and 10 μM) for 24 h, the HeLa cells that were stained with 10 mg mL^−1^ Hoechst 33258 for 60 min at 37 °C. And then gently washed the cell with 1× PBS for twice and photographed under an Olympus BH-2 fluorescence microscope (Nikon, Japan).^35^

### 2.12. TUNEL assay

Cells were seeded into confocal dishes (SPL, Korea) at a density of 20 000 cells per ml. After 12 h, cells were treated with 0.1% DMSO (as control) or compound PBQC (1, 5 and 10 μM) for 12 h, cell apoptosis rate was evaluated by terminal deoxynucleotidyl transferase-mediated dUTP nick-end labeling (TUNEL) assay.^33^ Briefly, cells were fixed with cold 4% paraformaldehyde for 25 min and incubated with 0.2% Triton X-100 for 5 min at room temperature after washed with 1× PBS three times, discarded the supernatant and then washed the plates with 1× PBS twice. Added 100 μL equilibration buffer into each hole for 10 min, then, poured off the supernatant and incubated cells with 50 μL rTdT incubation medium that was away from light for 60 min at 37 °C. After incubating for 1 h, we discarded the incubation medium and added 50 μL 2× SSC into the plates for 15 min to terminate the reaction. After washing cells with PBS three times, stained cells with 4′,6-diamidino-2-phenylindole (DAPI) for 5 min and observed and photographed by a confocal microscopy (Carl Zeiss, Germany), the excitation wavelength is 488 nm.

### 2.13. Western blot analysis

As described previously, cells were seeded into 6-well plates at a density of 20 000 cells per ml and treated with 0.1% DMSO or PBQC for 6 h, 12 h, 24 h, 48 h and washed twice with ice cold PBS, then lysed in protein lysis buffer (Shanghai Beyotime Co, China) that contained 0.5% SDS in 25 mM Tris–HCl, 4 mM EDTA, 100 mM NaCl, 1 mM PMSF, 10 μg mL^−1^ leupeptin and 10 μg mL^−1^ soybean trypsin inhibitor, pH = 7.5. The protein concentration of the cells was determined by the Bradford method. Before being loaded onto a 12% or 15% SDS polyacrylamide gel, equal amount of protein (30 μg) was added loading buffer and boiled. Following electrophoresis, the resolved protein was electrophoretically transferred to a Polyvinylidene Fluoride (PVDF) membrane (Millipore, MA, USA). The membrane was blocked with 5% non-fat milk in TBST (Tris buffer saline containing 0.5% Tween 20) for 1 h at room temperature. Subsequently, the membrane was probed with PARP antibody (1 : 2000) (Proteintech, USA), Nrf2 antibody (1 : 2000) (Proteintech, USA), p53 antibody (1 : 2000) (cell signaling, USA), Bcl-2 antibody (1 : 2000) (Proteintech, USA), cleaved caspase-3 antibody (1 : 2000) (cell signaling, USA), Bax antibody (1 : 2000) (Proteintech, USA) or anti-β-actin mouse monoclonal antibody (1 : 2000) (SIGMA, USA) overnight at 4 °C, then was washed twice with TBST, each time for 5 min. The membrane was subsequently incubated with HRP-conjugated goat anti-rabbit IgG (1 : 5000) (Beijing Dingguo Co, China) or polyclonal goat anti-mouse immunoglobulins/HRP (1 : 5000) (Beijing Dingguo Co, China) for 1 h at room temperature and then washed three times with TBST. Then the membrane was incubated with HRP substrate for 5 min and the fluorescence signal were detected with X-ray films. Intensity of the protein bands was quantified by Quantity-One software (Bio-Rad), analyzed by Image J software and normalized to loading controls.

### 2.14. Measurement of glutathione (GSH)

5,5′-Dithiobis-2-nitrobenzoic acid (DTNB) were used to measure the intracellular GSH by total glutathione assay kit (Beyotime Institute of Biotech, Jiangsu, China), following the manufacture's instruction.^36^ Briefly, HeLa cells were incubated with or without PBQC for 1, 3 and 6 h, cells were washed with PBS, and collected by centrifugation. Then, the sample was subjected to two rapid freeze–thaw cycles using liquid nitrogen and 37 °C water bath. The supernatant was taken for the determination of total glutathione. Simultaneously, 10 mM GSH stock solution was diluted with protein removal reagent S to 50 μM GSH solution. Then the 50 μM GSH solution was diluted to 25, 15, 10, 5, 2 μM GSH solution. 50, 25, 15, 10, 5, 2 μM GSH solutions were taking to do the standard curve. Finally, samples and standards were added to 96-well plate and the absorbance at A412 was measured on a NanoQuant microplate reader (TECAN). The GSH concentrations were determined by comparison with standards.

### 2.15. RNA interference

The transfection of a specific siRNA targeting Nrf2 was facilitated by HiperFect RNA interference reagent, according to the manufacturer's instructions. HeLa cells were seeded in 6-well plates, when the confluence reached 60%, HeLa cells were transfected with Nrf2 siRNA and scrambled siRNA as the negative control. After incubation with siRNAs for 6 h, the medium was substituted for normal culture medium, and the cells were then ready for subsequent experiments. The efficiency of silencing was evaluated by western blot assay.

### 2.16. Statistical analysis

Data were presented as means ± SEM from no less than three independent experiments and analyzed by SPSS (Statistical Package for the Social Sciences) software and Student's *t*-test. Pictures were processed with Adobe Photoshop software. The mean values were derived from at least three independent experiments. Differences at *p* < 0.05 were considered statistically significant.

## 3. Results

### 3.1. PBQC up regulated Nrf2 activity

There is a close relationship between the Nrf2–ARE signaling pathway and cancer cell apoptosis.^37,38^ For evaluating the effect of PBQC on Nrf2 activity, we used luciferase HeLa cells which can stably express firefly luciferase to immediately monitor the change of Nrf2 activity.^38^ Luciferase activity was detected after incubating cells with compound PBQC at indicated concentrations for specified time durations. The results showed a significant increase in Nrf2 activity, which suggested that PBQC activated Nrf2–ARE signaling. Furthermore, both 5 and 10 μM PBQC increased luciferase activity at 24 and 48 h as compared with controls. We were surprised to find that the Nrf2 activity was significantly increased and cell viability was relatively decreased with dose and time dependent. We observed the decrease in the number of luciferase HeLa cells after 12 h, and there was no significant decline before 12 h (Fig. 1B).

### 3.2. PBQC inhibited the growth of tumor cells while it did not affect the normal vascular endothelial cell viability

**Fig. 2:**
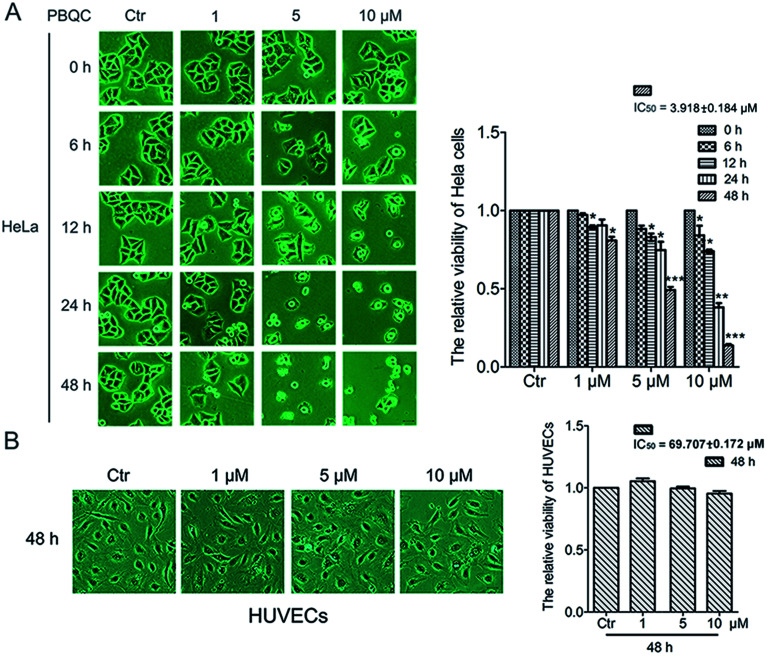
PBQC inhibited the growth of tumor cells but did not affect the normal vascular endothelial cell viability. (A) HeLa cells were exposed to compound PBQC at 1, 5 and 10 μM for 6, 12, 24 and 48 h, respectively. The cells obviously underwent morphological changes with the extension of time and the increase of PBQC concentration. The cell viability was measured by SRB assay. Bar as present 22 μM. (B) Effects of compound PBQC on human umbilical vein endothelial cell viability, the cells were treated with PBQC at the concentrations of 1, 5 and 10 μM or treated with DMSO 0.1% (v/v) (control) for 48 h. Cell viability was analyzed by SRB assay and illustrated in the labelled column. Set the control group activity to 1. Bar as present 22 μM. (*p < 0.05, **p < 0.01, ***p < 0.001, *vs.* control, *n* = 3).

In order to explore the biological effect of PBQC on tumor cells, we first observed the morphological changes of tumor cells, including HeLa, U87 and A549 cells, after treatment with 0.1% DMSO (control) or 1, 5 and 10 μM PBQC for 0, 6, 12, 24 and 48 h under a phase contrast microscope (Fig. 2A and S1†). We found that exposure of tumor cells to PBQC at 10 μM for 12, 24 or 48 h resulted in a serious decrease of cell viability. Meanwhile, the changes of morphology, vacuolization and shrinkage of tumor cells were obviously observed. For evaluating the inhibitory effects of PBQC on the growth of tumor cells, we carried out the SRB assay after treatment with PBQC of 1, 5 and 10 μM for 0, 6, 12, 24 and 48 h. The results showed that compound PBQC inhibited the growth of tumor cells in a dose dependent manner (Fig. 2A and S1†), the most potent inhibitory rate was 78% at 48 h with 10 μM PBQC in HeLa cells comparing with the control group. From the data, we can respectively calculate the half maximal inhibitory concentration of tumor cells for 48 h (HeLa, IC_50_ = 3.918 ± 0.184 μM, U87, IC_50_ = 9.212 ± 0.11 μM, A549, IC_50_ = 27.082 ± 0.153 μM). At the same time, we also treated human umbilical vascular endothelial cell with 0.1% DMSO (control) or 1, 5 and 10 μM PBQC for 48 h and observed the morphological changes and growth (Fig. 2B). PBQC had no significant effect on the normal cell viability. We can draw a conclusion that compound PBQC inhibits tumor cell growth, especially in HeLa cells, and does not affect the normal vascular endothelial cell growth. Therefore, in the next experiment, we focused on HeLa cells as a research model to study the inhibitory effect of PBQC on cancer.

### 3.3. PBQC up-regulated the expression level of Nrf2 in HeLa cells

**Fig. 3:**
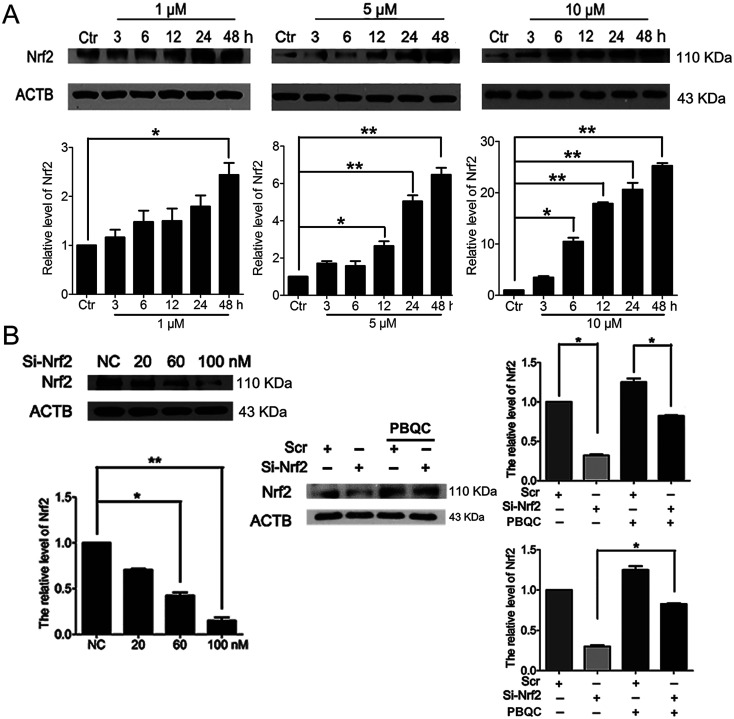
PBQC up regulated Nrf2 in HeLa cells. (A) Western blot analysis showed that Nrf2 level was up-regulated by PBQC prominently at 12 h and 24 h. (B) 100 nM si-Nrf2 dramatically decreased Nrf2 protein. And 10 μM PBQC prevented the decline of Nrf2. Set the control group activity to 1. (*p < 0.05, *vs.* control, *n* = 3).

In addition to Nrf2 activity, we discovered that PBQC raised the expression level of Nrf2 in HeLa cells. As shown in Fig. 3A, we pre-incubated HeLa cells with compound PBQC (1, 5 and 10 μM) for the specific time. PBQC treatment could increase the protein level of Nrf2. By performing Nrf2 silencing, we found that the knockdown of Nrf2 dramatically decreased Nrf2 protein in HeLa cells. But, the protein level of Nrf2 was obviously increased by PBQC in the Nrf2 silencing groups (Fig. 3B). It suggested that PBQC could increase the Nrf2 activity and up-regulate the expression level of Nrf2.

### 3.4. PBQC promoted Keap-1 glutathionylation and Nrf2 nuclear translocation

**Fig. 4:**
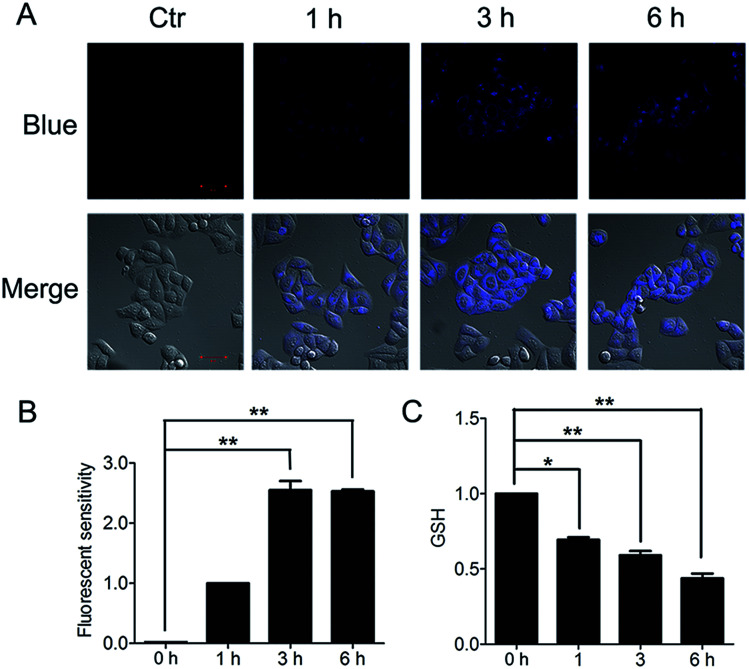
PBQC decreased the level of glutathione in Hela cells. (A) We treated the cells with 10 μM PBQC for 1, 3 and 6 h, then, detected the changes of the blue fluorescence by confocal microscopy. Bar as present 50 μM. (B) The blue fluorescence intensity was quantified by Image J. (C) We treated the cells with 10 μM PBQC for 1, 3 and 6 h, then, detected the level of glutathione by the total glutathione assay kit (Beyotime Institute of Biotech, Jiangsu, China), following the manufacture's instruction. (*p < 0.05, **p < 0.01, *vs.* control, *n* = 3).

**Fig. 5:**
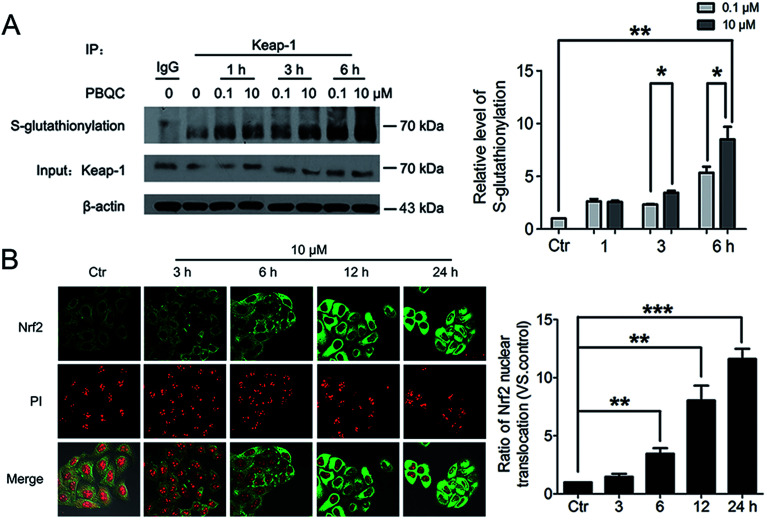
PBQC promoted Keap-1 glutathionylation and Nrf2 nuclear translocation. (A) WB analysis of co-IP of Keap-1 with glutathione antibody in HeLa cells treated with PBQC (0.1 and 10 μM) with 1% CS for 1, 3 and 6 h, quantification of co-immunoprecipitated Keap-1 levels. (B) Immunofluorescence assay of Nrf2 in HeLa cells incubated with PBQC (10 μM) for 3, 6, 12 and 24 h, nuclei were labeled with PI. Bar as present 50 μM. (*p < 0.05, **p < 0.01, *vs.* control, *n* = 3).

The important biological function of glutathione is to maintain the redox state of the protein thiol by the formation of protein–glutathione mixed disulfides. It was demonstrated that PBQC was able to detect GSH in living cells without interference from Cys and Hcy. This probe can quickly respond to the change of glutathione level when the body's redox reaction is unbalanced.^32^ Based on the sensing mechanism of PBQC toward GSH, we know the consumption of intracellular glutathione is increased (Fig. 4A and C), for the decrease in glutathione level led to Keap-1 *S*-glutathionylation increase.^39^ In order to evaluate the *S*-glutathionylation level of Keap-1 protein, we performed nonreducing western blot analysis by using a specific monoclonal anti-glutathione antibody that recognized *S*-glutathionylated protein. As shown in Fig. 5A, a different pattern of *S*-glutathionylation was observed when compared to the control group. Therefore, different concentrations of PBQC could cause varying degrees of Keap-1 *S*-glutathionylation. Next, immunofluorescence experiments were performed to demonstrate that Nrf2 distribution within the nucleus. The results showed that Nrf2 nuclear translocation was significantly increased (Fig. 5B).

### 3.5. PBQC up-regulated the expressions of anti-oxidant genes and decreased the intracellular ROS level

**Fig. 6:**
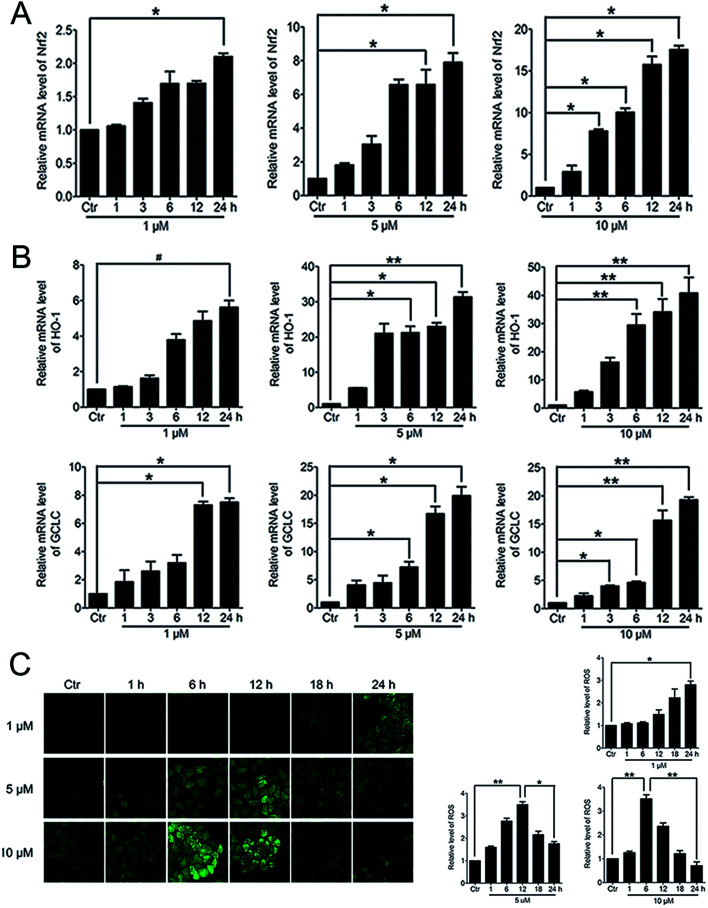
PBQC up regulated the expressions of anti-oxidant genes and decreased the intracellular ROS level. (A and B) RT-PCR analysis of mRNA levels of Nrf2, HO-1 and GCLC treated with PBQC (1, 5 and 10 μM) PBQC for indicated times. (C) Incubated HeLa cells with 0.1% DMSO (control) or PBQC at 1, 5 and 10 μM for 1, 6, 12, 18 and 24 h, then used 5 μM DCHF probe treated all cells for 30 min. The fluorescence intensity was observed by inverted fluorescence microscope (200×) and the exciting light of DCHF probe was blue light. ROS expression level was quantified by GraphPad Prism 5 software. Bar as present 22 μM. (*p < 0.05, **p < 0.01, *vs.* control, *n* = 3).

When Nrf2 get into the nucleus, the downstream anti-oxidant genes could be activated. RT-PCR analysis of mRNA levels of Nrf2, HO-1 and GCLC in HeLa cells treated with PBQC at 1, 5 and 10 μM for 1, 3, 6, 12 and 24 h was performed. As the results shown, compound PBQC significantly increased the mRNA levels of Nrf2 and the downstream anti-oxidant gene, HO-1 and GCLC (Fig. 6A and B). Next, we measured the intracellular level of ROS by using the fluorescent probe DCHF. We found that ROS level rose with the acceleration of GSH consumption, then, decreased with the increase of the anti-oxidant gene expression (Fig. 6C).

### 3.6. The varying degree of Nrf2 activation had different effects on the downstream Bcl-2 and Bax gene expressions

**Fig. 7:**
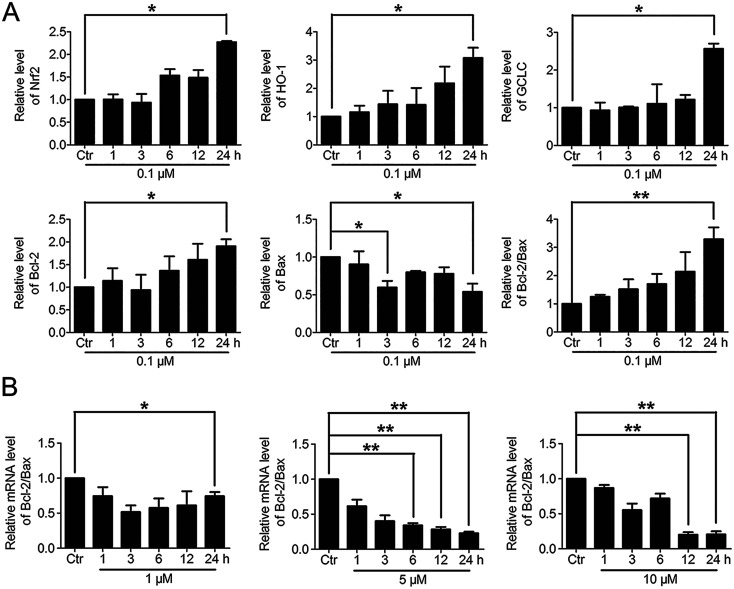
The varying degree of Nrf2 activation had different effects on the downstream Bcl-2 and Bax gene expressions. (A) RT-PCR analysis of mRNA levels of Nrf2, HO-1, GCLC, Bcl-2 and Bax in HeLa cells treated with PBQC (0.1 μM) for indicated times. (B) RT-PCR analysis of mRNA levels of Bcl-2 and Bax in HeLa cells treated with PBQC at 1, 5 and 10 μM for 1, 3, 6, 12 and 24 h, set the control group activity to 1. (#*p* > 0.05, *p < 0.05, **p < 0.01, *vs.* control, *n* = 3).

Based on the above findings that PBQC had the concentration-dependent roles in HeLa cells, we further detected the effects of PBQC at different concentrations on the mRNA levels of Nrf2, HO-1, GCLC, Bcl-2, Bax and β-actin by RT-PCR analysis. PBQC at 0.1 μM had no effect HeLa cell viability within 24 h (Fig. S2†), at the meantime, PBQC at the low concentration up regulated Bcl-2 and down regulated Bax, resulting in the increased ratio of Bcl-2 and Bax (Fig. 7A). But, at 1, 5 and 10 μM, PBQC decreased the ratio of Bcl-2 and Bax (Fig. 7B). The mRNA levels of Nrf2, HO-1 and GCLC were increased by PBQC both in the case of low concentrations and high concentrations.

### 3.7. Significant activation of Nrf2 by PBQC promoted NQO1 and p53 expressions

**Fig. 8:**
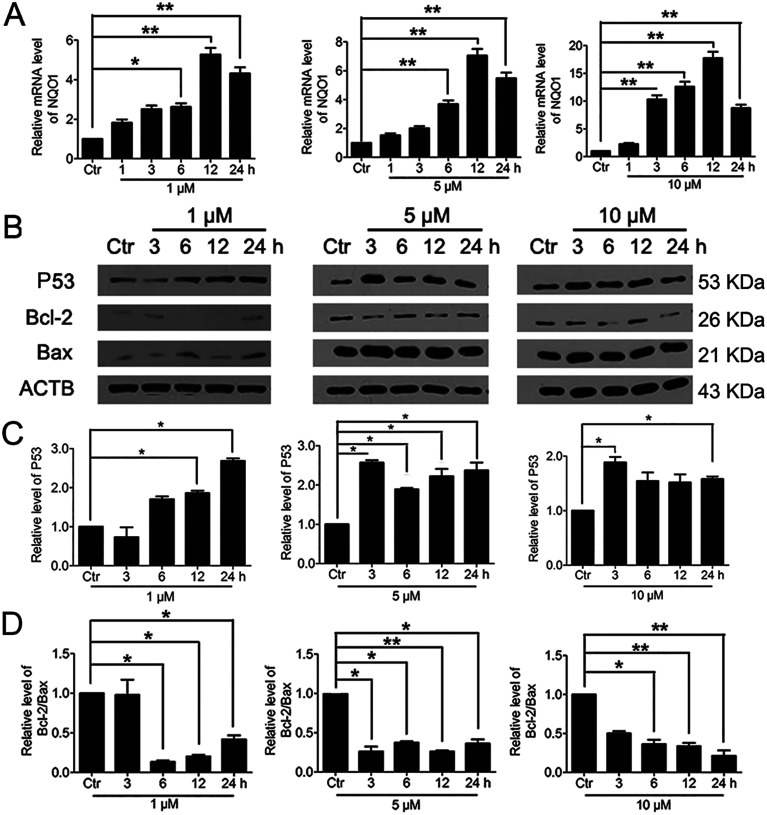
Significant activation of Nrf2 by PBQC promoted NQO1 and p53 expressions. (A) RT-PCR analysis of mRNA levels of NQO1 in HeLa cells treated with PBQC at 1, 5 and 10 μM for 1, 3, 6, 12 and 24 h. (B) Western blot analysis of the protein level of p53, Bcl-2, Bax and β-actin as a normalization control and quantitative statistics (C and D). HeLa cells were treated with 0.1% DMSO (control) or PBQC at 1, 5 and 10 μM for 3, 6, 12 and 24 h. We set the control group activity to 1. (*p < 0.05, **p < 0.01, *vs.* control, *n* = 3).

It was reported that significant increase of Nrf2 activity could also activate NQO1 and the downstream gene p53.^40^ Therefore, we examined the changes of NQO1 mRNA level and p53 protein level. The results showed that PBQC promoted the expression of NQO1 and p53 (Fig. 8A and B). It has been known that p53 is able to specifically inhibit the expression of Bcl-2 protein, but does not affect Bax protein expression.^41^ Finally, we investigated the expressions of Bcl-2 and Bax by western blotting. As shown in Fig. 8B–D, the level of anti-apoptotic protein Bcl-2 was decreased obviously, and the ratio of Bcl-2 and Bax significantly declined.

### 3.8. PBQC induced cancer cell apoptosis

**Fig. 9:**
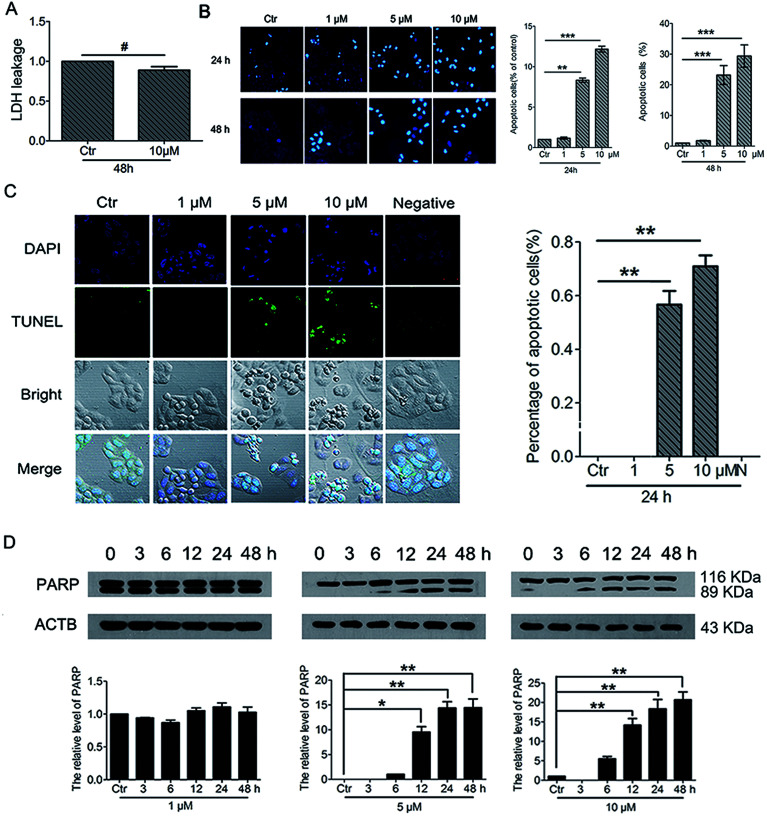
PBQC induced cancer cell apoptosis. (A) Effects of compound PBQC on LDH activity of HeLa cells. The culture medium was collected as samples for LDH assay after 48 h treatment at the concentration of 10 μM. Apoptosis-associated markers illustrated PBQC-accelerated cell dying in HeLa cells. (B) Hoechst 33258 staining testified that extra added PBQC (0, 1, 5 and 10 μM for 24 h) can increase the rate of HeLa cell-apoptosis. Microscope images (200×) were taken under a fluorescent microscope (Nikon). Apoptotic cells were quantitative. Bar as present 22 μM. (C) The changes of apoptosis of HeLa cells were observed by TUNEL assay. HeLa cells treated with 0.1% DMSO (control) or 1, 5 and 10 μM PBQC for 24 h were stained with TUNEL kit and confocal microscopic imaging was performed. Blue channel (405–640 nm), Ex = 405 nm; green channel (500–578 nm), Ex = 488 nm; statistics of the number of apoptosis cells. Bar as present 50 μM. (D) Western blot analysis of the protein levels of PARP and β-actin as a normalization control and quantitative statistics. HeLa cells were treated with 0.1% DMSO (control) or PBQC at 1, 5 and 10 μM for 6, 12, 24 and 48 h. (*p < 0.05, **p < 0.01, ***p < 0.001, *vs.* control, *n* = 3).

**Fig. 10:**
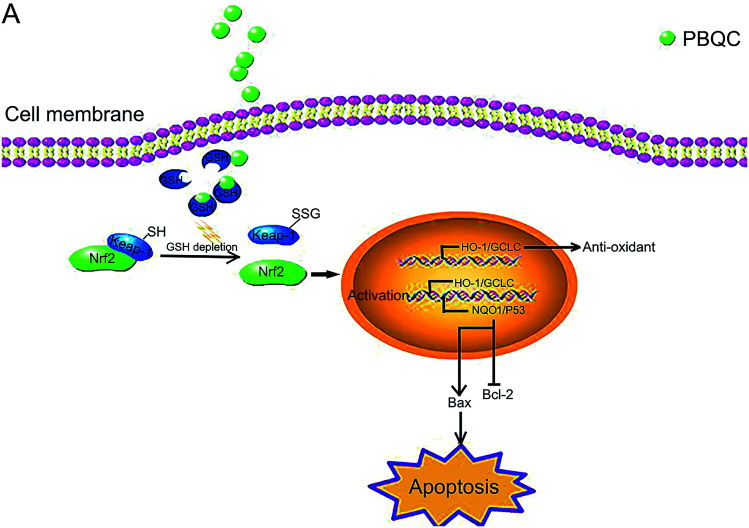
Schematic presentation of PBQC activating Nrf2 and inducing apoptosis. (A) PBQC treatment increases Keap-1 *S*-glutathionylation and promotes Nrf2 activity. Subsequently, the activated Nrf2 gets into the nucleus, anti-oxidative signaling pathway is activated. The significant activation of Nrf2 by PBQC up-regulates NQO1 and p53 and specifically inhibits the increase of Bcl-2 expression. The pro-apoptotic protein Bax is significantly increased. As a result, the cancer cells undergo apoptosis.

To further understand the cell fate after treatment with PBQC, we investigated the effects of PBQC on HeLa cell death. Firstly, to detect whether PBQC causes necrosis in HeLa cells or not, we measured the LDH activity in cell culture medium. We found that LDH release from HeLa cells treated with 10 μM PBQC for 48 h was no obvious difference comparing with the control group (Fig. 9A). Secondly, taking chromatin condensation and DNA fragmentation in the process of apoptosis into consideration, we stained DNA with Hoechst 33258 (Fig. 9B) and situ end labeling technique (TUNEL) of DNA fragmentation to evaluate apoptosis cells (Fig. 9C). We observed the obvious changes of the cell nucleus condensation. Thirdly, the western blotting assay showed that the level of cleaved PARP was elevated by PBQC (Fig. 9D). These results showed that PBQC actually induced HeLa cell apoptosis in a dose dependent manner (Fig. 10).

## 4. Discussion

In our previous work, we designed and synthesized PBQC which can sense glutathione (GSH) by PET process, discriminate GSH from cysteine and homocysteine, and specifically consume intracellular glutathione.^32^ The glutathione level is increased in many cancer cells,^4^ and interfering with intracellular glutathione of cancer cells can significantly promote the cell apoptosis.^7^ It was also reported that glutathione depletion induced the *S*-glutathionylation of Keap-1 protein and markedly increased Nrf2 activity.^27^ In this study, we found that PBQC could consume the intracellular glutathione. The decrease in glutathione level led to the increase of Keap-1 *S*-glutathionylation which promoted Nrf2 dissociation from Keap-1 and acquired the activity. PBQC promoted the *S*-glutathionylation of Keap-1 protein, activated Nrf2 and promoted cancer cell apoptosis. We provide a new Nrf2 activator that can promote *S*-glutathionylation of Keap-1 protein. Our data suggested that PBQC is a potential lead-compound for development of new anti-cancer drugs.

Importantly, we provide the new evidence that the varying degrees of Nrf2 activation induced by PBQC had different effects on the/its downstream gene expressions. In previous reports, higher concentrations of Nrf2 activators were used to demonstrate that they can give rise to tumor cells apoptosis, which came to the same conclusion here by using high PBQC concentrations.^26,42^ However, we also sought to explore the effects of PBQC at low concentrations, which were quite different from those at high concentrations. Therefore, we investigated the action of PBQC at different concentrations to provide a intriguing research idea. At the low concentrations, PBQC slightly activated Nrf2 and the downstream anti-oxidant genes, resulting in the increased ratio of Bcl-2/Bax and cell survival. But, at the high concentrations, PBQC markedly increased Nrf2 activity. Under this condition, the anti-oxidant signaling pathway was still activated, but the expression of the anti-apoptotic protein Bcl-2 was inhibited. Compared with Bcl-2 protein, the expression of pro-apoptotic protein Bax was significantly increased. As a result, the ratio of Bcl-2/Bax and the cell survival declined.

It was reported that dissociative Keap-1 also activated p53 protein,^40^ and p53 was able to specifically inhibit the expression of Bcl-2 protein, but not for Bax protein.^41^ To understand why PBQC at high concentrations inhibited the expression of anti-apoptotic protein Bcl-2, we further detected the expressions of the other Nrf2 downstream genes, including NQO1 and p53. In addition, increasing NQO1 activity inhibited the degradation of tumor suppressor protein p53.^43^ Consistently, we found that the significant activation of Nrf2 by PBQC up regulated NQO1 as well as p53 and specifically inhibited the increase of Bcl-2.

## Abbreviations

GSH: Glutathione
Nrf2: The nuclear factor erythroid-2 related factor 2
CNC: Cap-‘n’-collar
b-ZIP: Basic region-leucine zipper
ARE: The anti-oxidant response element
Keap1: The Kelch-like ECH-associated protein 1
PSH: A thiol group
PSSG: The *S*-glutathionylated protein
PBQC: 2-(7-(Diethylamino)-2-oxo-2*H*-chromen-3-yl)cyclohexa-2,5-diene-1,4-dione
ROS: Reactive oxygen species
DMSO: Dimethylsulfoxide
EDTA: Ethylene diamine tetraacetic acid
PMSF: Phenylmethanesulfonyl fluoride
SDS: Sodium dodecyl sulfate
LDH: Lactate dehydrogenase
DTNB: 5,5′-Dithiobis-(2-nitrobenzoic acid)
HO-1: Heme oxygenase-1
GCLC: Catalytic subunit of glutamylcysteine ligase
NQO1: NAD(P)H quinone dehydrogenase 1
PI: Propidium iodide
Bcl-2: B-cell lymphoma-2
Bax: Bcl-2 associated X protein
p53: The tumor protein p53
PBS: Phosphate buffered saline
TCA: Trichloroacetic acid
DAPI: 4′,6-Diamidino-2-phenylindole

## Conflicts of interest

The authors declare no conflict of interest.

## Supplementary Material

RA-008-C7RA11935F-s001
