# Supplementary material for: A small molecule targeting glutathione activates Nrf2 and inhibits cancer cell growth through promoting Keap-1 S-glutathionylation and inducing apoptosis
Source: RSC Adv. 2018 Jan 3;8(2):792–804. doi: 10.1039/c7ra11935f (PMC9076930; doi:10.1039/c7ra11935f)
Supplement: RA-008-C7RA11935F-s001 [file RA-008-C7RA11935F-s001.pdf]

Electronic Supplementary Material (ESI) for RSC Advances.
This journal is © The Royal Society of Chemistry 2017

1. Supplemental results:

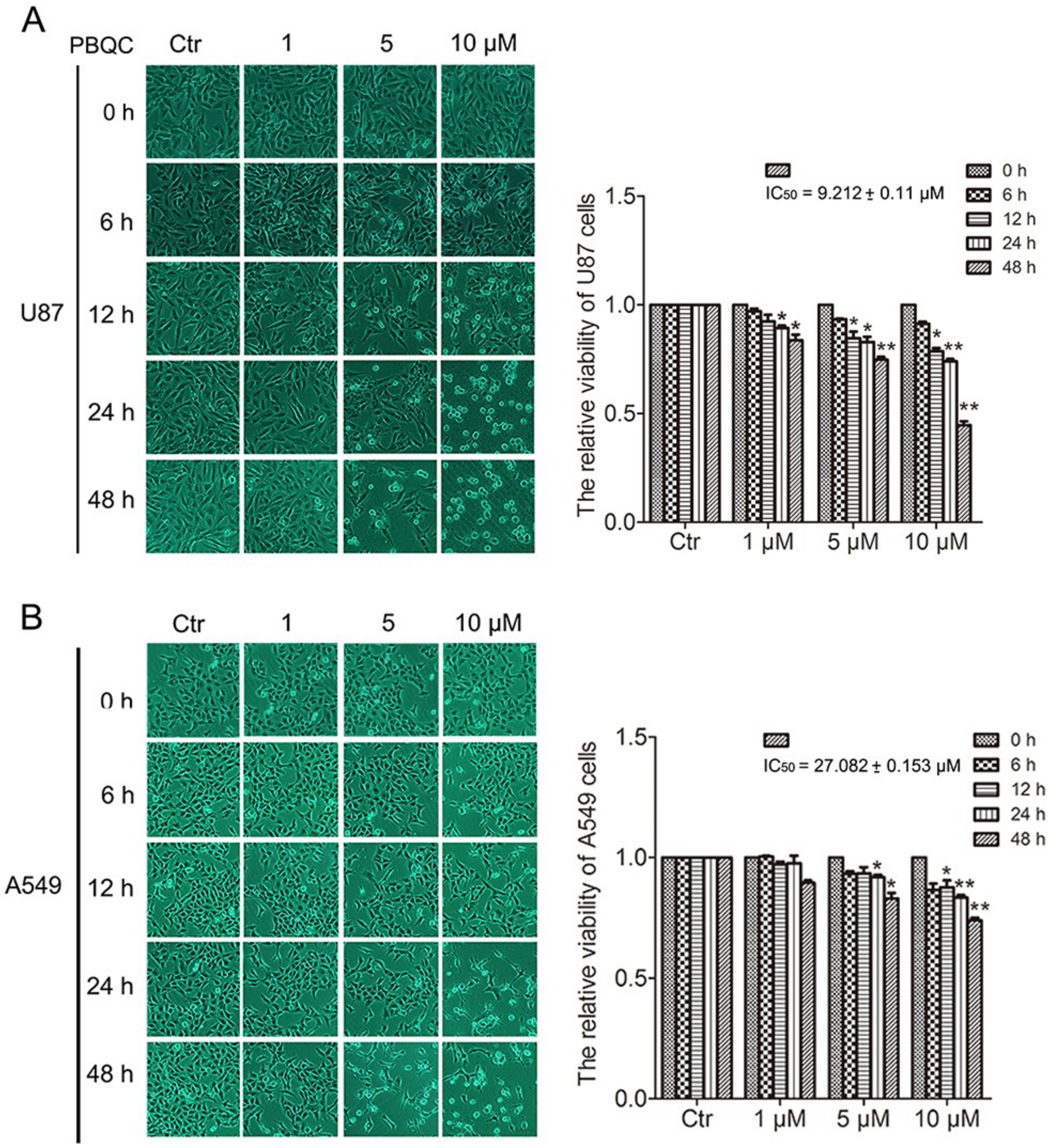

Figure S1. PBQC inhibited the growth of tumor cells

(A) U87 cells were exposed to compound PBQC at 1, 5 and 10 μM for 6, 12, 24 and 48 h, respectively. The cells obviously underwent morphological changes as the extension of time and the increase of PBQC concentration. The cell viability was measured by SRB assay. Bar as present 22 μM. (B) A549 cells were exposed to compound PBQC at 1, 5 and 10 μM for 6, 12, 24 and 48 h, respectively. The cells

obviously underwent morphological changes as the extension of time and the increase of PBQC concentration. The cell viability was measured by SRB assay. Bar as present 22 μM. (Data are presented as means ± SEM, *, $p < 0.05$, **, $p < 0.01$, VS. control, n=3).

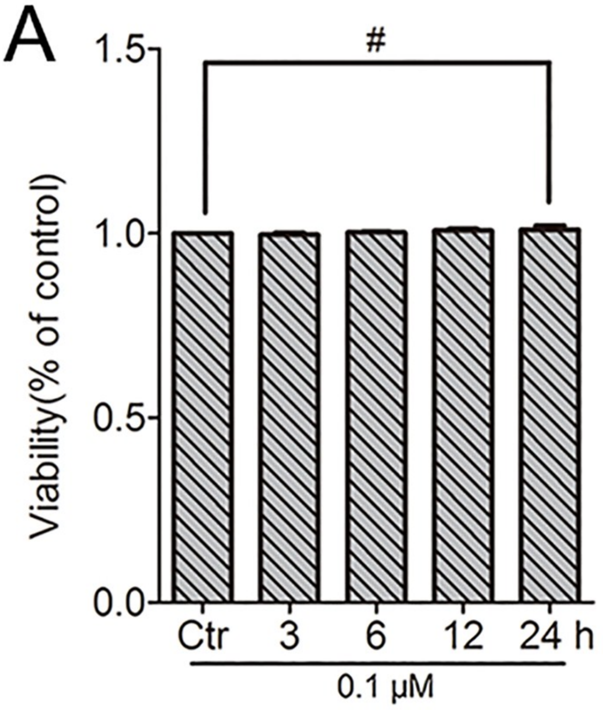

Figure S2. Low concentrations of PBQC did not inhibit the growth of Hela cells

(A) HeLa cell viability was measured by SRB assay at 0.1 μM PBQC for 3, 6, 12, 24 h. (Data are presented as means ± SEM, #, $p > 0.05$, VS. control, n=3).

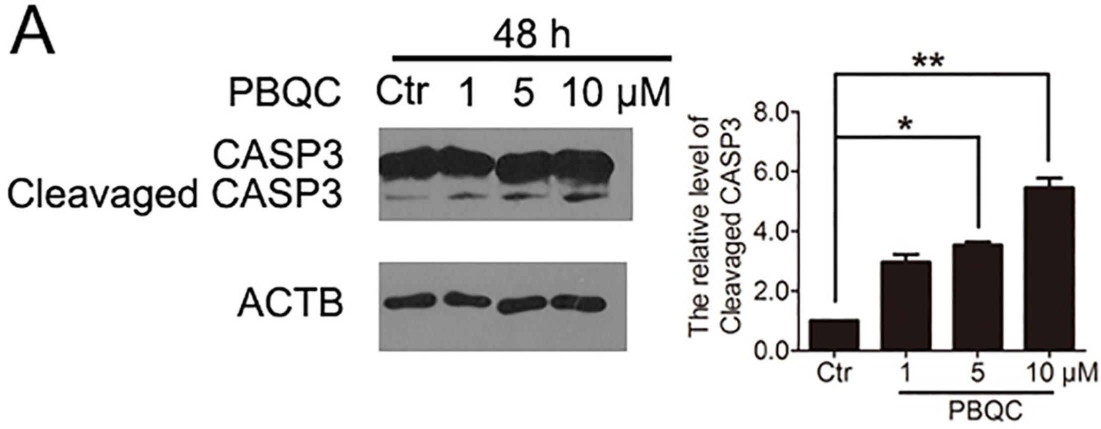

Figure S3. PBQC increased the protein level of cleavaged caspase 3.

(A) Western blot analysis showed that Cleavaged CASP3 level was up-regulated by 1, 5, 10 μM PBQC prominently at 48 h. Set the control group activity to 1. (*, $p < 0.05$, **, $p < 0.01$, VS. control, $n = 3$).
